# ‘*BJUI* Clinical Dilemma’: Recurrent high‐grade non‐muscle‐invasive bladder cancer in 2026

**DOI:** 10.1111/bju.70299

**Published:** 2026-04-28

**Authors:** Vignesh T. Packiam, Peter C. Black, James W. F. Catto, Saum Ghodoussipour, Max Kates, Ashish M. Kamat, Yair Lotan, Michael A. O’Donnell, Jeremy Y. C. Teoh, George N. Thalmann, Stephen B. Williams

**Affiliations:** ^1^ Rutgers Cancer Institute New Brunswick NJ USA; ^2^ Johns Hopkins University Baltimore MD USA; ^3^ The University of Texas MD Anderson Cancer Center Houston TX USA; ^4^ UT Southwestern Medical Center Dallas TX USA; ^5^ The University of Texas Medical Branch Galveston TX USA; ^6^ University of Iowa Iowa City IA USA; ^7^ University of British Columbia Vancouver British Colombia Canada; ^8^ University of Sheffield Sheffield UK; ^9^ The Chinese University of Hong Kong Hong Kong China; ^10^ University Hospital of Bern Bern Switzerland

**Abbreviations**
BST: bladder‐sparing therapy
CG: cretostimogene grenadenorepvec
CHAI: Computational Histology Artificial Intelligence
CIS: carcinoma *in* situ
CR: complete response
CSS: cancer‐specific survival
EAU: European Association of Urology
FDA: United States Food and Drug Administration
Gem/Doce: gemcitabine and docetaxel
GSC: Genomic Subtype Classifier
HG: high grade
IBCG: International Bladder Cancer Group
IL‐12: interleukin 12
(N)MIBC: (non‐)muscle‐invasive bladder cancer
NAI: nogapendekin alfa inbakicept
NCCN: National Comprehensive Cancer Network
PD‐1: programmed death 1
PFS: progression‐free survival
RC: radical cystectomy
RFS: recurrence‐free survival
TRAE: treatment‐related adverse event
TURBT: transurethral resection of bladder tumour

## Introduction

Following transurethral resection of bladder tumour (TURBT), high‐grade (HG) non‐muscle‐invasive bladder cancer (NMIBC) is traditionally first treated with BCG. Unfortunately, recurrence and progression have been long‐standing problems. There has been a welcome explosion of salvage therapies, which have also resulted in new challenges of optimal treatment selection and sequencing. In this context, recurrence following a single induction or multiple courses of BCG are contemporary clinical dilemmas.

The present ‘*BJUI* Clinical Dilemma’ presents a structured format to address the common clinical presentation of recurrent HG NMIBC through a case vignette approach. Following the vignette, leading experts will provide brief commentary, explaining rationale for management strategies, novel considerations, and ongoing research. This approach encourages a balanced, expert‐driven discussion on alternative strategies, offering readers insights into decision‐making processes for common, yet nuanced cases in urological practice.

## Case Vignette

A 74‐year‐old man presented to his primary care physician with painless gross haematuria. A CT urogram was obtained that demonstrated a 3.5‐cm left bladder mass and was otherwise unremarkable. He has a 21 pack‐year smoking history and takes medications for hypertension and hyperlipidaemia. He has no relevant family history and denies weight loss, flank pain, or constitutional symptoms. He urinates one to two times per night and otherwise denies bothersome LUTS. Physical examination was unremarkable. A full blood count, comprehensive metabolic panel, urine analysis, and chest X‐ray were normal.

The patient was referred to a local urologist and TURBT was performed. The left papillary tumour was completely resected. A satellite red patch was resected from the trigone. Pathology returned as HG T1 (muscle present) and carcinoma *in situ* (CIS; trigone) urothelial carcinoma. A repeat TURBT was benign. A month after the resection, the patient was initiated on induction full‐dose BCG, which he tolerated well, apart from mild dysuria and frequency. At his first surveillance cystoscopy two 1‐cm papillary tumours were visualised in the trigone. A complete TURBT was performed and pathology returned as HG Ta urothelial carcinoma.

The patient presents to you for a second opinion as to what would be his best course of action.

### BCG Resistance: Definitions and Need for Simplicity? (Catto/Kamat)

Terminology surrounding BCG ‘failure’ has evolved significantly, yet inconsistencies in application complicate clinical decision‐making and trial design. The framework adopted by the United States Food and Drug Administration (FDA) in 2018 was based on expert consensus opinion [1, 2], and defined ‘BCG*‐*unresponsive’ disease as persistent or recurrent HG NMIBC despite adequate BCG—specifically, HG Ta/T1 within 6 months or CIS within 12 months of the last instillation after receiving at least five of six induction and two of three maintenance or re‐induction doses. There is a third category of BCG*‐*unresponsive disease that includes HG T1 at first evaluation after induction BCG alone. Recognising the large population outside these criteria, the 2022 International Bladder Cancer Group (IBCG) consensus introduced the term ‘BCG‐exposed’, encompassing: ‘BCG‐resistant’ disease (persistent HG Ta/CIS at 3 months post‐induction), delayed relapse after inadequate BCG (recurrence within 24 months following induction‐only therapy), and delayed relapse after adequate BCG (recurrence beyond the unresponsive window but within 24 months) [3]. These distinctions are critical for harmonising research endpoints, yet from a clinical standpoint, simplicity is key. The practicing urologist should focus on timing, pattern, and grade of recurrence rather than navigating a taxonomy of overlapping terms. Ultimately, pragmatic definitions, anchored in disease biology and therapeutic response, will serve both scientific rigour and clinical relevance, ensuring that patients are neither denied potentially curative intravesical therapy nor subjected to ineffective salvage attempts.

### Initial Options (Ghodoussipour)

This patient, appropriately treated with induction BCG after an initial diagnosis of HG T1/CIS NMIBC, now presents with recurrent HG Ta disease. Based on current FDA and consensus definitions, this is considered BCG‐exposed/BCG‐resistant disease. This patient is likely to benefit from repeat BCG, but management at this juncture should incorporate shared decision‐making that includes discussion of all options including radical cystectomy (RC), repeat BCG, intravesical chemotherapy and clinical trials.

Radical cystectomy plays an important role in patients with NMIBC who are at the highest risk of progression, as cure is more commonly achieved before progression to MIBC [4, 5]. Historical data shows that early RC following a high‐risk recurrence has been associated with a lower incidence of progression to MIBC and improved cancer‐specific survival (CSS) [6, 7]. However, RC carries significant morbidity and quality of life changes that most patients prefer to avoid when safe alternatives exist [8]. Importantly, this patient has persistent HG Ta disease, which reflects neither stage nor grade progression. Contemporary evidence shows that patients with papillary‐only recurrence after BCG can be effectively managed with bladder‐sparing therapy (BST) with a 1‐year recurrence‐free survival (RFS) of 44% and progression‐free survival (PFS) of 89% at 18 months [9]. Should further recurrence occur, novel salvage therapies approved in the BCG‐unresponsive setting are associated with relatively low rates of progression (0–17%) and RC‐free survival exceeding 80% in most series [10].

Repeat induction BCG remains an effective salvage option after a single induction course, with better responses seen after delayed relapse beyond 12 months [11]. In patients with earlier recurrence, such as this case, recent data show encouraging outcomes, including 5‐year PFS of 77%, CSS of 95% and RFS >75% with additional BCG alone [12, 13].

While BCG has demonstrated superiority over single‐agent chemotherapy in Phase III trials, there is emerging evidence to consider alternative agents in the BCG‐exposed space [14]. A Phase II trial suggested gemcitabine may improve 2‐year RFS compared with repeat BCG, although PFS was similar (19% vs 3% and 77% vs 75%, respectively) [15]. Combined chemotherapy with sequential intravesical gemcitabine and docetaxel (Gem/Doce) has yielded intriguing results, with 1‐year HG RFS of 65% in patients with BCG‐unresponsive disease and 85–92% in patients with treatment‐naïve disease, although most evidence remains retrospective [16, 17, 18].

Although most novel agents and drug‐delivery systems have been evaluated in the BCG‐unresponsive or treatment‐naïve settings, many are now being actively investigated in the BCG‐exposed space [10]. These may soon offer additional bladder‐preserving options to minimise risk of progression or need for RC.

Given the absence of grade or stage progression in this case, the availability of effective intravesical salvage strategies, and the current lack of validated biomarkers to guide early switching to chemotherapy, the preferred next step for this patient is repeat induction with BCG. Intravesical chemotherapy, novel agents (on or off trial), or RC can be reserved as subsequent options should further recurrence occur.

### Role of Biomarkers (Teoh/Williams)

Various biomarkers have been developed to provide prognostic and predictive information for Ta/T1 NMIBC, yet these are rarely used in clinical practice [19, 20]. In this particular context, a biomarker that can predict the treatment response from re‐induction BCG therapy could be useful. Recently, the Computational Histology Artificial Intelligence (CHAI) platform has been developed, which quantifies histological features from digital whole‐slide images, and generates biomarkers capable of predicting the risk of recurrence and progression in patients with high‐risk NMIBC following TURBT and intravesical BCG treatment [21]. Based on a retrospective cohort of 269 patients with Ta HG NMIBC, the presence of the CHAI biomarker was significantly associated with HG RFS in multivariate analyses after adjusting for AUA and European Association of Urology (EAU) risk groups [22]. The CHAI biomarker was also significantly associated with PFS, in which disease progression was defined as the development of MIBC or nodal/distant metastasis [22]. In another study, the role of CHAI biomarker in predicting response to re‐induction BCG was investigated [23]. A total of 52 patients previously treated with BCG who developed recurrent high‐risk NMIBC undergoing repeat BCG induction were included [23]. The study showed that the CHAI‐biomarker present group had a significantly shorter time to HG recurrence compared to the CHAI‐biomarker absent group. At 6 months, the HG RFS rates were 70% in the CHAI‐biomarker present group and 22% in the biomarker‐absent group [23]. While data are still relatively limited, the CHAI platform can provide important information on the decision of intravesical BCG therapy vs alternatives in patients with high‐risk NMIBC [24, 25].

Another biomarker which could be helpful in this case is the Decipher Bladder Genomic Subtype Classifier (GSC). Being able to identify molecular subtypes that are associated with poor prognosis could identify the need for early RC [26, 27]. In a cohort of 343 patients with MIBC, GSC was able to predict four consensus molecular subtypes with 73% accuracy [27]. Its clinical significance was also validated, showing that luminal tumours had the best overall survival with or without neoadjuvant chemotherapy, and claudin‐low tumours were associated with poor overall survival irrespective of treatment regimen [27]. In another cohort of 206 patients with clinical T1–T2 HG bladder cancer who underwent RC, patients with luminal tumours were found to have lower rates of upstaging to non‐organ‐confined disease compared to non‐luminal tumours [28]. The use of GSC could also help identify patients with neuroendocrine‐like subtype, which has been associated with significantly worse outcomes [29].

## Case Vignette Continued

The patient elected to get re‐induction full strength BCG. At his next surveillance cystoscopy a red patch was seen on the right lateral wall and voided cytology returns positive for urothelial carcinoma. Blue light cystoscopy and TURBT is performed, which demonstrates two additional fluorescent lesions. Pathology demonstrated multifocal CIS. The patient has nocturia three to four times and daytime urinary frequency, occasional dysuria. He asks you about options and your recommendation.

## Management Options

1. Radical cystectomy (Thalmann)
2. Gemcitabine and docetaxel (O’Donnell)
3. United States Food and Drug Administration‐approved agents: pembrolizumab, nadofaragene firadenovec, nogapendekin alfa inbakicept (NAI)/BCG, gemcitabine intravesical system (TAR‐200) (Lotan/Black)
4. Clinical trials (Packiam)

### Radical Cystectomy (Thalmann)

In this patient with symptomatic early HG recurrence and multifocal CIS, RC with urinary diversion is the favoured treatment option. According to the EAU NMIBC Guidelines, ‘treatments other than radical cystectomy should be considered oncologically inferior in patients with BCG‐unresponsive disease’. A delay in RC may lead to decreased CSS. It is well known that patients in whom RC is performed before progression to MIBC fare better with 5‐year cancer‐free survival rates >80% [30, 31, 32, 33].

With such early recurrence and multifocal CIS, care should be taken to exclude upper tract disease as a site of recurrence [34]. Once this is excluded the option of RC needs to be discussed in depth with the patient, also the issue of urinary diversion and the different alternatives. With this pathological constellation in younger patients, sexual organ‐sparing in women and bilateral nerve‐sparing with preservation of the seminal vesicles and the dorsal prostate capsule in men can be offered with no oncological risk [35, 36].

### Gemcitabine and docetaxel (O’Donnell)

Historically, for HG BCG‐unresponsive disease, RC was the preferred treatment, but it can be argued that this was primarily due to the lack of available effective alternative options. Realising that the expected 1‐year progression rate is <10% and that the mortality rate does not reach 2.5% (typical mortality rate for RC) until beyond 1.5 years [37], plus the incredible recent advances in later stage therapy [38], recent expert consensus by the IBCG is that there is a relatively safe window of opportunity of at least a year to consider BST [39]. Indeed, a large multi‐institutional study retrospectively comparing various alternatives to upfront RC found that both cancer‐specific and overall survival were statistically similar at over 5 years [37]. Furthermore, Gem/Doce was the highest performing treatment regimen among the BST therapies examined, which consisted of mostly older regimens such as repeat BCG [40, 41].

To date, most of the data supporting Gem/Doce for BCG‐unresponsive disease has come from retrospective studies including a 2020 study involving 10 academic institutions where the 1‐ and 2‐year HG RFS for the 71 patients with recurrent CIS was 60% and 50%, respectively [16]. Furthermore, the 2‐year progression rate was only 7% with a CSS of 96% [16]. These favourable outcomes were reproduced in three independent retrospective studies [42, 43, 44] along with a first, albeit small, prospective study [45]. Moreover, Gem/Doce treatment has been very well tolerated with <5% dropout rate, <1% Grade 3 treatment‐related adverse events (TRAEs) and shown to be durable with over two thirds of 2‐year responders remaining cancer‐free out to 5 years, while maintaining a low 18% risk of progression, and high 75% RC‐free rate and 91% CSS [46]. For these reasons Gem/Doce is now endorsed by the AUA, National Comprehensive Cancer Network (NCCN), EAU, and IBCG as an option for BCG‐unresponsive patients either unfit or refusing RC [39, 47, 48, 49]. It is likely that constant vigilance including enhanced surveillance protocols for both local and early extravesical recurrences contributed to these favourable results [50].

However, most patients in BST programmes eventually fail with HG disease and it is unclear whether the net result is simply a delay in eventual RC or cascading trials of other emerging BST regimens until untoward progression or metastasis ensue. At least one such successful sequencing of alternative intravesical doublet chemotherapies has been reported [51]. Noteworthy was the observation that despite jumping onto up to three subsequent rescue therapies over the median 86‐month follow‐up, neither RFS nor PFS declined significantly across the different rescue therapies resulting in a 7‐year PFS of 75%, RC‐free survival of 68%, and CSS of 83% [51]. Ongoing research suggests combining Gem/Doce with anti‐programmed death 1 (PD‐1)/programmed death‐ligand 1 (PD‐L1) agents might provide even more superior outcomes [52].

### Current FDA‐Approved Agents (Lotan/Black)

In 2018 the FDA provided a registration pathway for single‐arm trials to treat patients with BCG‐unresponsive CIS with or without papillary disease [1, 2, 53]. This has resulted in multiple approved agents for the treatment of these patients including pembrolizumab (2020), nadofaragene firadenovec (2022), NAI/BCG (2024) and gemcitabine intravesical system (TAR‐200; 2025) [54, 55, 56, 57]. These new drugs work primarily by stimulating the immune system (pembrolizumab, nadofaragene firadenovec, NAI/BCG) or through intravesical sustained release of chemotherapy (gemcitabine intravesical system). The trials that led to approval had similar inclusion criteria of patients with BCG‐unresponsive CIS based on FDA definition with or without papillary tumours. However, the trials varied in their approach regarding use of mandatory biopsies and ability to retreat patients with persistent disease at the time of first evaluation. For example, the trials for pembrolizumab, nadofaragene firadenovec, and gemcitabine intravesical system did not allow retreatment but NAI/BCG patients could get retreatment. The main endpoints for the trials were anytime complete response (CR) rates at 3 or 6 months and durability of response. The anytime CR rates for pembrolizumab, nadofaragene firadenovec, NAI/BCG and gemcitabine intravesical system were 41% (95% CI 31–51%), 51% (95% CI 41–61%), 62% (95% CI 51–73%) and 74.5% (95% CI 65.4–82.4%), respectively [54, 55, 56, 57]. The 12‐month CR rate for these agents was 19%, 24%, 45% (including post re‐induction) and 46%, respectively [54, 55, 56, 57].

There are several challenges for clinicians when determining best steps for a patient with BCG‐unresponsive CIS ± papillary disease. The pivotal trials leading to approval of the above agents were non‐randomised so direct comparison of efficacy is not reliable. The trials also included small cohorts of ~100 patients with different baseline rates of prior treatments and courses of BCG. CIS is notoriously difficult to detect with white light, and the variable use of mandatory biopsies impacted the ultimate CR rates for different treatments. The differential use of re‐induction therapy further confounds any comparison across trials.

Nonetheless, clinicians still need to guide patients to select their next treatment. There are no biomarkers currently to personalise the decision between treatments. As such, a shared decision‐making process is reasonable, whereby the available data on efficacy and toxicity. as well as cost and convenience of application, are presented to the patient. Pembrolizumab offers the distinct disadvantage of systemic toxicity that is only is rarely observed with intravesical treatments. As such, use of pembrolizumab is primarily confined to patients with very poor bladder capacity or poor tolerance of intravesical agents who refuse RC, or after exhaustion of other options. Among the intravesical agents, there are some distinct differences. The gemcitabine intravesical system is inserted into the bladder every 3 weeks for 24 weeks of induction therapy and requires a cystoscopic procedure to retrieve the indwelling device each time after the first instillation. Nadofaragene firadenovec is instilled only once every 3 months, but the agent requires storage at ≤−60°C and needs 3 h to defrost. NAI is given with BCG so the availability of BCG can be a factor while there is a mechanism to apply for recombinant BCG through an access trial. These newer agents all carry considerable cost, so the financial implications need to be considered as well, especially in comparison to intravesical Gem/Doce [58].

### Clinical Trials and Emerging Therapies (Packiam)

While off‐label and recently FDA‐approved therapies for NMIBC exist, there remains a search for more effective and well‐tolerated treatments. There is a wide array of ongoing investigation for novel agents and drug delivery systems for BCG‐unresponsive disease [10]. Cretostimogene grenadenorepvec (CG), which currently holds a FDA Fast Track and Breakthrough Therapy designation, seems closest to gaining FDA approval in the near future. CG is a conditionally replicating adenovirus engineered to lyse tumour cells with Rb‐pathway defects and promote expression granulocyte‐macrophage colony‐stimulating factor (GM‐CSF) to stimulate anti‐tumour immunity [59, 60]. The BOND‐003 trial (ClinicalTrials.gov identifier: NCT04452591) recently evaluated CG monotherapy in 112 patients with BCG‐unresponsive CIS with or without papillary disease, finding anytime CR rate of 75.5%, 1‐year CR rate of 46%, 2‐year CR rate of 42%, with no Grade ≥3 TRAEs [61]. CG in combination with pembrolizumab has demonstrated 1‐year CR rate of 57.1%, although there were 14.3% Grade ≥3 TRAEs [62]. Therefore, CG monotherapy seems preferable.

There are a variety of other therapies under evaluation [10]. The LEGEND trial (NCT04752722) is investigating detalimogene voraplasmid (EG‐70), which is an intravesical non‐viral gene therapy utilising a plasmid to express interleukin 12 (IL‐12) and retinoic acid‐inducible gene I (RIG‐I) agonists. A recent interim update from the trial demonstrated 71% anytime CR rate, 6‐month CR rate of 47% and no Grade ≥3 TRAEs [63]. The ADVANCED‐2 trial (NCT05951179) is assessing TARA‐002, an attenuated *Streptococcus pyogenes* formulation, in a mixed population of patients with BCG‐naïve unresponsive disease [64]. NDV‐01 is a sustained‐release reformulation of Gem/Doce that has also reported results in a heterogenous cohort of BCG‐naïve unresponsive disease with promising early results [65]. There is a recently activated trial evaluating MVR‐T3011, which is a novel herpes simplex virus (HSV)‐based oncolytic immunotherapy engineered to co‐express IL‐12 and PD‐1 inhibition, without need for bladder pre‐wash [66]. There are many other ongoing investigations in the BCG‐unresponsive, BCG‐exposed, BCG‐naïve spaces, as well as in intermediate‐risk disease, making this an exciting time to witness developments of therapies that can help our patients [10]. A necessary next innovation will be to understand how to best use precision medicine and biomarker directed therapy and offer therapeutics to more directly limit the development of iatrogenic LUTS and end‐stage bladder.

### Guideline Recommendations and Opportunities (Kates)

The NCCN, AUA, and EAU guidelines for NMIBC share a common principle: in patients with BCG‐unresponsive disease, RC remains the treatment offering the most durable cancer control, yet is a life altering, potentially morbid intervention [47, 48, 49]. Each guideline also emphasises the importance of shared decision‐making in managing patients with recurrence after adequate BCG, recognising the complex trade‐offs between efficacy, morbidity, and quality of life. Yet, despite this guidance, there is a pressing need for more robust comparative data to inform patient‐centred decisions.

The recently completed CISTO trial (NCT03933826) sought to address this gap by comparing second‐ and third‐line intravesical or systemic therapies with RC for patients with BCG‐unresponsive or relapsing NMIBC [67]. That study demonstrated favourable patient‐reported outcomes and higher satisfaction among patients undergoing RC compared to those pursuing additional bladder‐preserving therapies. However, beyond these data, clinicians lack head‐to‐head comparisons across the expanding array of approved agents. The single‐arm trials leading to recent FDA approvals used heterogeneous eligibility criteria, endpoints, and re‐induction protocols, limiting cross‐trial interpretation. Furthermore, many of these new agents carry substantial financial burden, often costing hundreds of thousands of dollars annually, adding another dimension to treatment decision‐making.

To truly realise the promise of novel therapies for BCG‐unresponsive NMIBC, we must strengthen the evidence base guiding shared decisions. This includes comparative trials of second‐line agents, ongoing biomarker discovery using urine, tissue, and artificial intelligence (AI)‐based tools to optimise treatment sequencing, and a deeper understanding of financial toxicity relative to clinical benefit. Only through such efforts can urologists and patients make informed choices, rather than merely educated ones.

## Disclosure of Interests

Vignesh T. Packiam: Consultant for Valar Labs, Photocure, Ferring, and Veracyte. Speaker for Johnson and Johnson, Veracyte, and Photocure. Peter C. Black: Advisory board or equivalent with AbbVie, Astellas, AstraZeneca, Aura, Bayer, BMS, CG Oncology, Combat, EMD‐Serono, EnGene, Ferring, Janssen, Merck, Nonagen, NanOlogy, Nanobot, Pfizer, Photocure, Prokarium, Sumitomo, TerSera, Tolmar, and Verity. Holds a shared patent with Veracyte. James W. F. Catto: Has received reimbursement for consultancy from AstraZeneca, Ferring, Roche, and Janssen; speaker fees from Bristol Myers Squibb, Merck Sharp and Dohme, Janssen, Astellas, Pfizer, and Roche; honoraria for membership in advisory boards from Ferring, Roche, Bristol Myers Squibb, and Janssen; and research funding from Roche. Saum Ghodoussipour: Receives research support from Janssen, Pangea Laboratory, Veracyte, and CG Oncology. Serves as an ad hoc consultant/speaker for Urogen, Janssen, Ferring, Intuitive Surgical, Karl Storz, Natera, and Early is Good. Max Kates: Consultant/Advisor for Relmada, Aura, Nanology, Merck, Pfizer, AstraZeneca, and Urogen. Ashish M. Kamat: Has received research funding from FKD Therapies (now Ferring), the Patient‐Centered Outcomes Research Institute (PCORI), Photocure, Seagen, EnGene, Arquer Diagnostics, and Southwest Oncology Group (SWOG). Has also received advisory board or consulting fees from Astellas Pharma, Atonco Pharma, Biological Dynamics, Bristol‐Myers Squibb, CG Oncology, Cystotech, Eisai, EnGene, Ferring, Genentech, Imagin Medical, ImmunityBio, Imvax, Incyte, Janssen, Medac, Merck, Nonagen Bioscience, Pfizer, Photocure, Protara Therapeutics, Roche, Seagen, Sesen Bio (now Carisma), Theralase, Urogen Pharma, US Biotest, Valar Labs, and Vivet Therapeutics. Holds a joint patent with MD Anderson Cancer Center for CyPRIT (Cytokine Predictors of Response to Intravesical Therapy). Holds/recently held leadership or fiduciary roles in the International Bladder Cancer Group (IBCG), European Urology Oncology, the Journal of Urology, UroToday, AUA, and World Bladder Cancer Patient Coalition. Yair Lotan: Consultant for Photocure, Astra‐Zeneca, Merck, Fergene, Nucleix, Ambu, Seattle Genetics, Virtuoso Surgical, Stimit, Urogen, Vessi Medical, CAPs Medical, Nonagen, Aura Biosciences, Inc., Convergent Genomics, Pacific Edge, Pfizer, Phinomics Inc., CG Oncology, Uroviu, Promis Diagnostics, Valar Labs, Uroessentials, NRx Pharmaceuticals, Vesica Health, Janssen, Immunity Bio, Trigone Pharma, and Relmada. Michael A. O'Donnell: Consultant for Fidia Farmaceuticals and Theralase. Consultant and Investigator for Pfizer and Urogen. Jeremy Y. C. Teoh: Receives honoraria/lecture fees from Astellas, Boston Scientific, Combat Medical, Ferring, Ipsen, Janssen, Olympus, and Sanofi. Serves on consultancy/advisory boards for Astellas, Aulea Medical, CMR Robotics, Combat Medical, EQT, Ferring, Illumicell AI, Janssen, MRI PRO, Phase Scientific, and Procept BioRobotics. Receives research grants from Baxter, Bristol‐Myers Squibb, Ferring, Janssen, Merck Sharp & Dohme, Storz, and Olympus. George N. Thalmann: Discloses affiliation with OnconiX Ltd. Stephen B. Williams: Consultant for Photocure, Janssen, Merck, Valar Labs, and Optheras.
